# Study on Effect and Mechanism of *β*-Aminobutyric Acid on Mango Anthracnose Caused by *Colletotrichum gloeosporioides*

**DOI:** 10.3390/foods14173061

**Published:** 2025-08-30

**Authors:** Cuiping Pan, Jing Wang, Yiyue Wang, Huaiyu Yuan, Ying Liu, Ke Li, Lian Tao, Yongqing Zhu, Huajia Li

**Affiliations:** 1 Institute of Agro-Products Processing Science and Technology/Institute of Food Nutrition and Health, Sichuan Academy of Agriculture Sciences, Chengdu 610066, China; pcp399@163.com (C.P.); wangjing11991199@163.com (J.W.); wangyiyue0903@163.com (Y.W.); yuanhuaiyu53@163.com (H.Y.); liuy6321@scsaas.cn (Y.L.); like2341@126.com (K.L.); zhuyongqing68@sina.com (Y.Z.); 2 Peanut Research Institute, Nanchong Academy of Agricultural Sciences, Nanchong 637000, China; 3 Horticulture Research Institute, Sichuan Academy of Agriculture Sciences, Chengdu 610066, China; tao_lian@163.com

**Keywords:** mango fruit, postharvest, induced resistance, enzyme activities, mechanism

## Abstract

Anthracnose is one of the most serious postharvest diseases that can manifest in mango. The mechanism and inhibitory effects of *β*-aminobutyric acid (BABA) on anthracnose in harvested mango fruit were investigated. The “Guifei” fruits were pretreated with different concentrations of 25, 50, 75, and 100 mmol/L BABA, with 0 mmol/L BABA as the control, and inoculated with *Colletotrichum gloeosporioides*. The results showed that 50 mmol/L BABA treatment significantly reduced the incidence of anthracnose and inhibited the growth of lesions in mango. It significantly increased the activities of antioxidant enzymes such as superoxide dismutase (SOD), catalase (CAT), and peroxidase (POD), while reducing the O_2_^−^ production rate and H_2_O_2_ content. In addition, the DPPH radical scavenging capacity was enhanced, the content of disease-resistance-related compounds, including total phenols and total flavonoids, increased, and the expression levels of defense-related genes such as PAL, GLU, CHI, and PR1 were upregulated, elevating the activity of phenylalanine ammonia-lyase (PAL) and pathogenesis-related proteins such as chitinase (CHI) and *β*-1,3-glucanase (GLU). In conclusion, BABA treatment significantly enhances mango fruit resistance to anthracnose via synergistically activating the antioxidant defense system, promoting the accumulation of disease-resistant compounds, and regulating defense-related gene expression. These findings provide a theoretical foundation for developing eco-friendly strategies to control postharvest diseases in mango.

## 1. Introduction

Mango fruit is known for its unique flavor, thin skin, and juicy texture. It contains a variety of nutrients, including vitamins, proteins, dietary fiber, and minerals, as well as bioactive compounds contributing to its high nutritional and commercial value [1,2,3,4,5]. However, as a typical respiratory leap-type fruit, mango is particularly susceptible to postharvest decay during transportation, storage, and sales, especially since the harvest season coincides with periods of high temperatures and heavy rainfall. These conditions make the fruit vulnerable to pathogenic infections, leading to the deterioration in quality and spoilage [6,7,8], which significantly hinders the development of the mango fruit industry.

Anthracnose, caused by the pathogen *Colletotrichum gloeosporioides*, is one of the most serious postharvest diseases to appear in mango. Postharvest losses attributable to this disease are estimated to range from 30% to 60% [9]. Currently, the industry predominantly depends on chemical fungicides for preservation. However, their application is increasingly limited by the rise of pathogen resistance, environmental contamination, and risks to human health. Therefore, an eco-friendly, safe, and effective preservation method is urgently needed in the mango industry. In recent years, the development of technologies aimed at inducing disease resistance has emerged as a prominent research focus in the field of postharvest disease management in fruits. It is regarded as the most promising method to replace chemical fungicides for controlling disease. The technology of inducing disease resistance involves treating fruit products with biological and non-biological-inducing factors to enhance their immunity and indirectly reduce infection of pathogenic fungus, extending their storage life. As such, it has great application potential.

BABA is a plant-derived, small-molecule, non-protein amino acid that has demonstrated significant potential in controlling plant diseases. It not only enhances plant stress tolerance but also induces disease resistance, improving the plant’s ability to resist pathogen infections [10,11,12,13,14,15]. Plants develop a complex defense system during growth and development. It has been shown that exogenous BABA can induce multiple defense responses and mitigate damage caused by disease as well as cold temperatures in plants. BABA treatment enhances resistance to *Penicillium chrysanthemum* in citrus and soft rot in peach through the activation of defense-related enzymes [16,17]. Genes related to resistance to grape berry disease were significantly upregulated to induce systemic defense after inoculation with Staphylococcus griseus and BABA treatment [18]. These results suggest that the inhibitory effect of BABA on pathogen fungus growth and plant signals contributes to fruit-induced resistance. However, previous research on BABA’s priming defense against postharvest anthracnose in mango only concentrated on the investigation of physiological parameters during the prime stage of fruit [16,19]; therefore, the mechanism behind BABA-induced mango resistance is unknown.

In the present study, “Guifei” mango fruit was utilized to assess the effects of BABA treatments in controlling the incidence of postharvest anthracnose, the antioxidant capacity of fruits, the activities of disease-resistant enzymes, and the relative expression levels of defense-related protein genes. We aimed to explore the inhibitory effect of BABA on mango anthracnose and possible mechanism behind this while providing a theoretical basis for the application of BABA in the postharvest preservation of mango fruits.

## 2. Materials and Methods

### 2.1. Materials

“Guifei” mangoes were harvested in May 2023 from the orchard of farmers in Zongfa Town, Renhe District, Panzhihua City, Sichuan Province. They were transported to the laboratory within 12 h of picking. Fruits with consistent size, coloration, and ripeness, devoid of pests, diseases, and mechanical damage, were selected and spread on the experimental bench, with field heat dispersed by natural wind.

*Colletotrichum gloeosporioides* was purchased from Beijing Baiou Bowei Biotechnology Co., Ltd., (Beijing, China) and the strain number was bio-81580. The activated *Colletotrichum gloeosporioides* was cultured on PDA medium for 8 days (28 °C), the spores of *Colletotrichum gloeosporioides* were washed out with sterile water, and the concentration of spore suspension was adjusted to 1.0 × 105 CFU/mL via a blood cell counting plate.

### 2.2. BABA Treatment and Inoculation

For mango fruits with a dispersed field heat, the surface of the samples were first wiped to remove the gum and then soaked in 2% sodium hypochlorite solution for 5 min to disinfect the surface of the fruit. It was then dried naturally, before two holes were punched with a diameter of around 5 mm and a depth of 3 mm into the equatorial symmetry of the fruit using a sterile perforator. The fruit segments were then randomly allocated into five groups, each group comprised three replicates, and each replicate contained 30 fruits. According to the groups, 20 μL of BABA solution (25, 50, 75, 100 mmol/L) was injected into the perforated parts of the fruits, with 0 mmol/L BABA injection as the control, and the fruits were placed at 25 ± 0.5 °C for 6 h. Subsequently, the perforated parts were inoculated with 20 μL of *Colletotrichum gloeosporioides* spore suspension. After completion of the treatment, the fruits were stored at 25 ± 0.5 °C and 80% to 90% RH.

### 2.3. Determination of Disease Spot Diameter and Incidence of Mango

Fruit spot diameter and incidence were recorded every 3 d after storage. Each treatment comprised three replicates, and each replicate contained 15 fruits. The size of lesions at the inoculation site of the mango was measured with vernier calipers, and samples with lesions larger than 5 mm in diameter were considered diseased fruits.

### 2.4. Sample Collection

Enzyme activities and metabolites were measured in fruits treated with a concentration of 50 mM BABA. Samples were collected every 3 d during the storage period, and healthy fruit flesh tissues were cut, quickly mixed, and snap-frozen with liquid nitrogen. The frozen samples were broken into powder and stored at −80 °C in an ultra-low-temperature freezer for subsequent analysis. Sampling for each treatment was performed with three replicates, each containing 3 fruits.

### 2.5. Determination of Total Phenol and Total Flavonoid Content

Sample pretreatment: A total of 1 g of a frozen material was homogenized with 5 mL of pre-chilled ethanol, followed by centrifugation at 4 °C and 13,000× *g* for 20 min. Then, the supernatant was taken and set aside at a low temperature. Total phenol content was quantified according to the Folin–Ciocalteu method [20]. A standard curve was constructed using gallic acid (GA), and the results were expressed in mg/g. Total flavonoid content was quantified using the NaNO_2_-Al (NO_3_)_3_-NaOH colorimetric method, the curve was constructed using rutin, and results were expressed in mg/g.

### 2.6. Measurement of Active Oxygen Content

The H_2_O_2_ content was determined using a Solarbio micro assay kit, and the O_2_^−^ generation rate was determined according to the “Guidelines for Postharvest Physiology and Biochemistry Experiments on Fruits and Vegetables”.

### 2.7. Measurement of DPPH Free Radical Scavenging Capacity

The DPPH free radical scavenging capacity was determined according to method of Baliyan et al. [21], with some modification. We placed 1 g of the frozen sample in 5 mL of pre-cooled ethanol; ground it into a pulp in an ice bath; and extracted it overnight at 4 °C. We then centrifuged it at 4 °C and 13,000× *g* for 10 min before taking 0.2 mL of the supernatant and adding 3.8 mL of 0.12 mmol/L DPPH solution. The sample was thoroughly shaken and mixed before the reaction was performed. The absorbance was measured at 517 nm after the samples were kept in the dark for 30 min. The supernatant was also replaced by anhydrous ethanol as a blank control.

### 2.8. Determination of Enzyme Activities in Mango Fruits

SOD, CAT, POD, PAL, GLU, and CHI activities were determined using Beijing Solarbio Technology Co., Ltd., (Beijing, China) a superoxide dismutase activity kit, catalase activity kit, peroxidase activity kit, phenylalanine aminotransferase activity kit, *β*-1,3-glucanase activity kit, and chitinase activity kit.

### 2.9. Determination of Relative Expressions of Related Defense Genes

Relative gene expression was assessed by real-time quantitative PCR (RT-qPCR). Consumables with sample RNA were treated with RNAase inhibitors and autoclaved, except for those already provided in the kit. In total, 80–100 mg of mango samples was weighed, quick-frozen in a mortar with liquid nitrogen, and then quickly ground until there was no visible tissue debris, and total RNA extraction was performed according to Fruit-mate™ for RNA Purification instructions. The cDNA preparation was performed according to PrimeScript™ II 1st Strand cDNA Synthesis Kit instructions, using no more than 1000 ng of total RNA in a 10 μL reaction system. The RT-qPCR reaction system was carried out following the manufacturer’s protocol for the AceQ^®^ qPCR SYBR^®^ Green Master Mix. The detection platform was RotorGene 3000, and the amount of cDNA template did not exceed 100 ng in a 20 μL reaction system. The primer sequences are shown in Table 1. Program settings: starting temperature at 95 °C, 5 min, 1 cycle; denaturation 95 °C, 15 s; annealing 60 °C, 30 s; and extension 72 °C, 50 s, 40 cycles. The relative expression of genes was calculated using the 2^-∆∆Ct^ algorithm [22].

The genes for defense-related proteins in mango [23], namely, PAL gene (NC_058137.1), GLU gene (DQ366708.1), CHI gene (NC_058149.1), and PR1 gene (JF271926.1), were selected to determine the relative gene expression.

### 2.10. Data Analysis

All experimental indicators were performed with three replicates, and the measurement results were expressed as the mean ± error. Excel 2019 was used for statistics and plotting the experimental data. A two-way ANOVA was conducted with SPSS software (v26.0 SPSS Inc., Chicago, IL, USA) to assess the effects of treatment. Differences between means were examined by Duncan’s multiple range test, with a significance threshold set at *p* < 0.05.

## 3. Results

### 3.1. Effect of BABA on Anthracnose Prevention and Treatment in Mango Fruit

**Figure 1:**
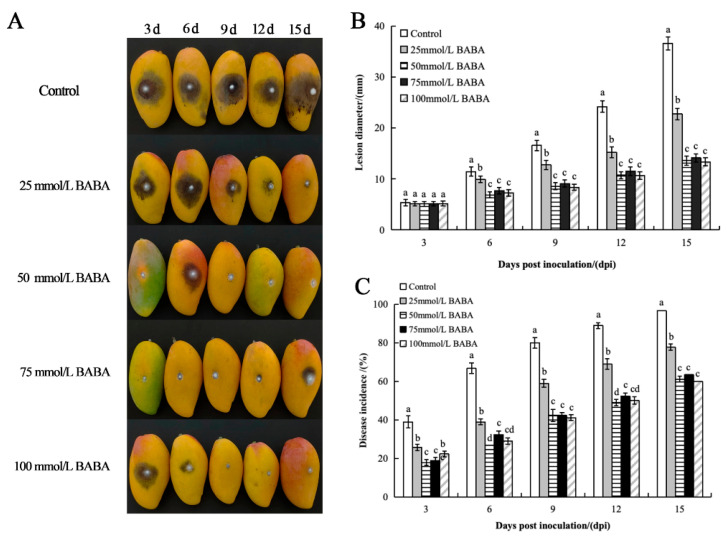
Effects of different concentrations of BABA treatments on the disease symptoms (**A**), lesion diameter (**B**), and incidence (**C**) of mango fruits. Error bars represent the standard deviation of the mean; different letters above columns at the same time point indicate statistically significant differences (*t*-test, *p* < 0.05).

Mango fruits were inoculated with different concentrations of BABA solution and anthrax bacillus. The incidence of anthracnose and the diameter of the disease spots increased with time during storage. Six days after inoculation, the diameter of the lesion and the incidence rate of the mangoes in the control group were significantly (*p* < 0.05) higher than those in all BABA treatment groups. Fifteen days after inoculation, all control fruits were diseased (Figure 1A), and the diameter of the lesion reached 36.58 mm (Figure 1B). whereas incidence rate in BABA-treated fruits was 60–76.67% (Figure 1C). The diameter of the lesion was 13.37–22.75 mm, while the diameter of the lesion in the treatment group was only 36.55–62.19% that of the control group. BABA effectively inhibited the occurrence of mango anthracnose and the increase in the lesion diameter. Among the various concentrations, the treatment effect of BABA at 50 mM was the most effective.

### 3.2. The Effect of BABA Treatment on the Accumulation of Total Phenols and Total Flavonoids in Mango Fruit

**Figure 2:**
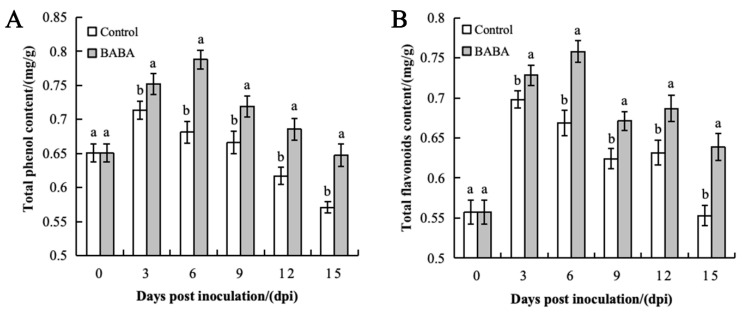
Effect of BABA treatment on total phenols (**A**) and total flavonoids (**B**) in mango. Error bars represent the standard deviation of the mean; different letters above columns at the same time point indicate statistically significant differences (*t*-test, *p* < 0.05).

The total phenol content of mango generally exhibited a similar trend of initial increase followed by a decrease in both control and treatment group, however, the total phenol content of the treatment group remained consistently and significantly higher throughout the experimental period. The total phenol content of the fruits in the control group reached its peak on the third day of storage at 0.714 mg/g before slowly decreasing. The total phenol content in the BABA treatment group continued to rise until the sixth day before reaching its peak at 0.788 mg/g. Although it gradually decreased afterwards, the treatment group’s phenol content was consistently and significantly superior to those of the control group during the latter stages of storage. At its peak, the total phenol content of the treatment group was 1.10 times that of the control group (Figure 2A).

The dynamics of the total flavonoid content in mango fruit basically paralleled that of the total phenolic content (Figure 2B). On day 3 of storage, the total flavonoid content in the control group significantly increased (*p* < 0.05) and reached its peak at 0.698 mg/g before slowly decreasing. The total flavonoid content in the treatment group rose rapidly and reached its maximum value of 0.758 mg/g on the sixth day, which was significantly higher than that of the control group (*p* < 0.05). At its peak, the total flavonoid content of the treatment group was 1.09 times that of the control group.

In conclusion, BABA treatment not only induced an increase in the total phenolic and flavonoid content in mango fruit but also effectively delayed the decline of these compounds in inoculated samples during the later stages of storage.

### 3.3. Effect of BABA Treatment on H_2_O_2_ Content and O_2_^−^ Production Rate in Mango Fruit

**Figure 3:**
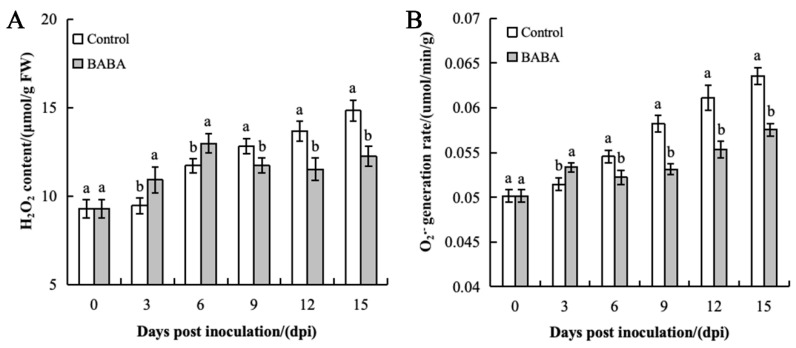
Effect of BABA treatment on H_2_O_2_ content (**A**) and O_2_^−^ production rate (**B**). Error bars represent the standard deviation of the mean; different letters above columns at the same time point indicate statistically significant differences (*t*-test, *p* < 0.05).

During the storage period, the H_2_O_2_ content and O_2_^−^ production rate in the control group exhibited a continuously increasing trend, whereas the treatment group displayed distinct variation characteristics (Figure 3). Specifically, the H_2_O_2_ content and O_2_^−^ production rate in the treatment group peaked on the sixth and third days, respectively, before stabilizing. The control group accumulated significantly more H_2_O_2_ and exhibited a higher O_2_^−^ production rate than the treatment group during the later storage phase (*p* < 0.05). On the 15th day of storage, the H_2_O_2_ content and O_2_^−^ production rate in the control group reached 14.821 μmol/g and 0.064 μmol/(min·g), respectively, while those in the treatment group were reduced by 17.63% and 9.38%, respectively. These results indicate that BABA effectively inhibits ROS accumulation in mango fruits during the later storage period, thereby mitigating oxidative damage.

### 3.4. Effect of BABA Treatment on DPPH Radical Scavenging Capacity in Mango Fruit

**Figure 4:**
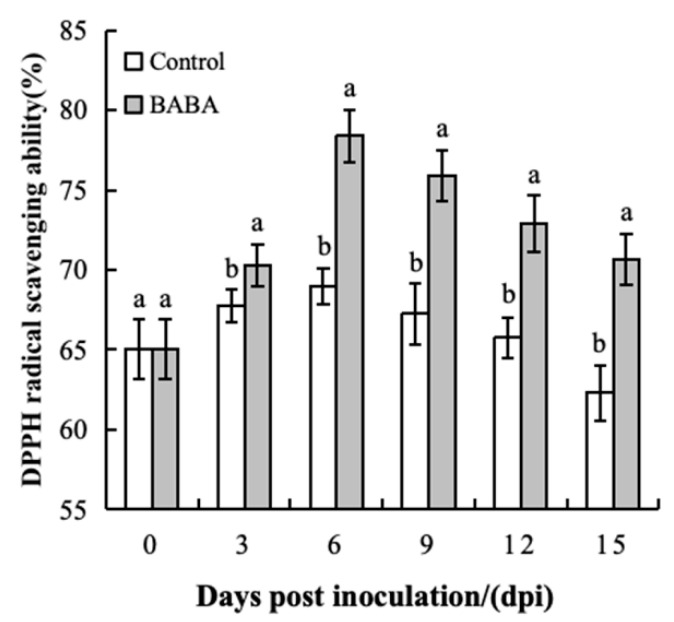
The effect of BABA treatment on the scavenging capacity of DPPH radicals. Error bars represent the standard deviation of the mean; different letters above columns at the same time point indicate statistically significant differences (*t*-test, *p* < 0.05).

During storage, the DPPH free radical scavenging rate of treated mangoes was significantly higher than that of the control group (*p* < 0.05). After six days of storage, the DPPH free radical scavenging rate was highest in the treatment group, reaching a value 1.16 times that of the control group. Throughout the storage period, the DPPH free radical scavenging rate of the BABA treatment group was significantly higher than that of the control group. These results demonstrate that BABA treatment can effectively and persistently enhance the DPPH radical scavenging capacity of mango (Figure 4).

### 3.5. Effect of BABA Treatment on SOD, CAT, and POD Activities in Mango Fruit

**Figure 5:**
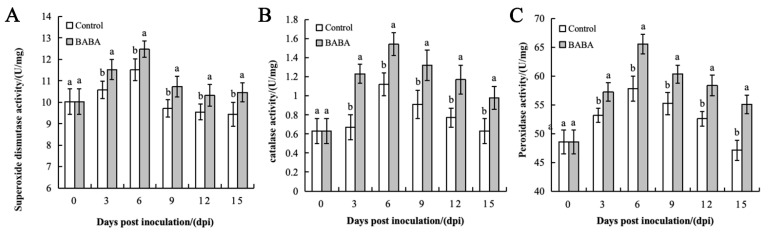
The effect of BABA treatment on the activities of SOD (**A**), CAT (**B**), and POD (**C**). Error bars represent the standard deviation of the mean; different letters above columns at the same time point indicate statistically significant differences (*t*-test, *p* < 0.05).

The activities of SOD, CAT, and POD in both the treatment group and the control group showed an initial increase followed by a decreasing trend, and both reached their peak on the sixth day. At this time, the rates of SOD, CAT, and POD in the treatment group were 12.2 U/mg, 1.5 U/mg, and 65.5 U/mg, respectively, while those in the control group were 11.5 U/mg, 1.1 U/mg, and 57 U/mg (Figure 5). Throughout the storage process, the activities of these three enzymes in the treatment group were consistently higher relative to the control. This indicates that BABA treatment can induce an increase in various antioxidant enzyme activities as well as the antioxidant capacity of mango.

### 3.6. Effect of BABA Treatment on PAL, GLU, and CHI Activities in Mango Fruit

**Figure 6:**
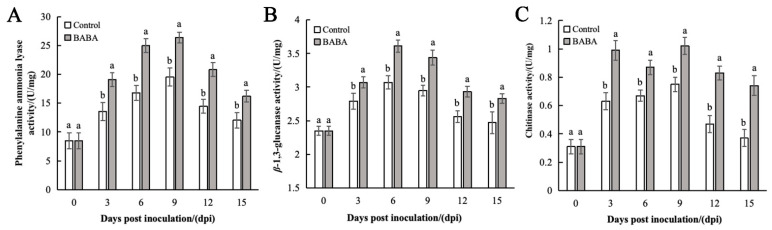
The effect of BABA treatment on the activities of PAL (**A**), GLU (**B**), and CHI (**C**). Error bars represent the standard deviation of the mean; different letters above columns at the same time point indicate statistically significant differences (*t*-test, *p* < 0.05).

During the storage period, the PAL activity in both the treatment and control groups exhibited an initial rise and subsequent decrease; however, the enzyme activity in treatment groups remained significantly higher than that in control group (*p* < 0.01) (Figure 6A). Specifically, after 3 days of storage, the PAL activity in the control group increased slowly; in contrast, PAL activity in the treatment groups rapidly increased and was 47.64% higher than that of the control group. On the ninth day after inoculation, the PAL activity in treatment groups peaked, reaching 1.51 times that of the control group at the same time point.

The GLU activity in the control group exhibited an initial increase followed by a decrease during storage, whereas the GLU activity in the BABA-treated group remained significantly higher (*p* < 0.05). The GLU activity in the treated group peaked on day 6, showing a 14.66% increase compared to the control, indicating that BABA treatment effectively induced GLU activity in mango (Figure 6B). The CHI activity of mango treated with BABA increased rapidly and reached its maximum on the ninth day. Subsequently, the activity decreased but remained significantly higher than that of the control group (*p* < 0.05) (Figure 6C). These results demonstrate that BABA can enhance mango’s resistance to pathogen infection by significantly elevating the activity of defense-related enzymes.

### 3.7. Correlation Analysis of the Incidence Rate of Mango Anthracnose, Lesion Diameter, Membrane Lipid Peroxidation, Antioxidant Enzyme Activity, and the Activity of Disease-Related Proteins

Inoculation was performed with *Colletotrichum gloeosporioides*, and the correlation analysis results between the fruit incidence rate and lesion diameter of mango fruits and membrane lipid peroxidation indicators, resistant compound levels, and disease-resistant enzyme activities in mango are shown in Table 2. The incidence rate and lesion diameter are significantly positively correlated with the O_2_^−^ production rate, significantly negatively correlated with the total phenol and flavonoid contents, and significantly negatively correlated with the activities of SOD and CAT. The incidence rate and lesion diameter are also significantly negatively correlated with the activities of GLU and CHI.

### 3.8. The Effect of BABA Treatment on the Expression of Defense-Related Genes in Mango Fruit

**Figure 7:**
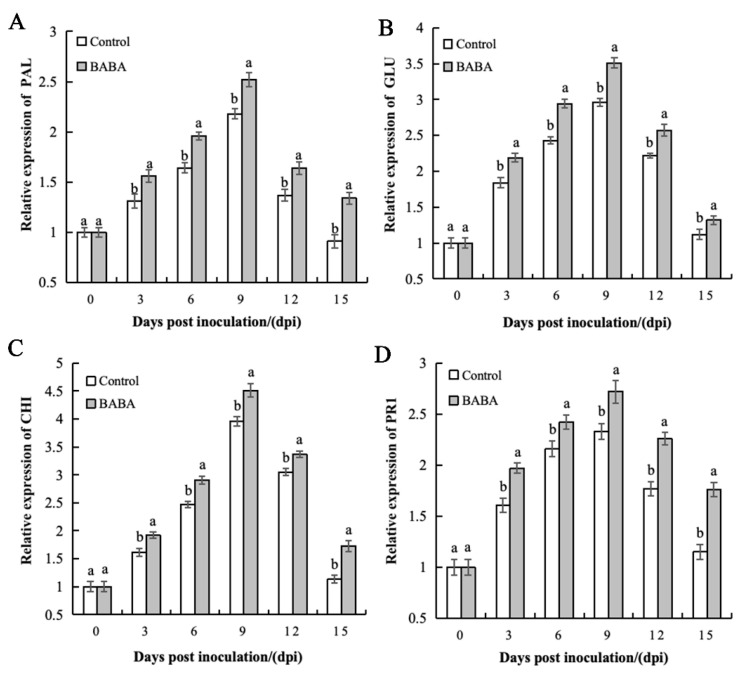
The effect of BABA treatment on the gene expressions of defense-related proteins PAL (**A**), GLU (**B**), CHI (**C**), and PR1 (**D**). Error bars represent the standard deviation of the mean; different letters above columns at the same time point indicate statistically significant differences (*t*-test, *p* < 0.05).

In comparison to the control, BABA treatment resulted in a substantial upregulation of PAL, GLU, CHI, and PR1 genes, which maintained high levels of expression. On the ninth day after inoculation (midway through the experiment), the expression levels of four defense genes in the treatment group reached their peak values. Among them, the expression levels of PAL, GLU, CHI, and PR1 were significantly upregulated by 1.55 times, 2.51 times, 3.47 times, and 1.77 times, respectively, relative to the control group. The results indicate that BABA treatment enhances mango’s resistance to anthracnose by persistently activating the expression of disease-resistance-related genes (PAL, GLU, CHI, and PR1), thereby boosting the activity of defense-related enzymes such as PAL, GLU, and CHI. This may indicate a key molecular mechanism underlying the improved resistance of mangoes receiving treatment (Figure 7).

## 4. Discussion

Disease resistance is an important mechanism that plants have evolved over time [20]. BABA is an environmentally friendly plant inducer and is considered an important signaling molecule in regulating plant-induced disease resistance. It is widely involved in the defense response of plants against pathogenic fungus and is widely used to preserve fruits and vegetables postharvest [24]. The optimal concentration of BABA treatment varies for different fruits. Wang et al. [25] determined that 10–500 mmol/L BABA treatment not only induces disease resistance in strawberries but can also improve the storage quality of these fruits. Elsherbiny et al. [26] reported that postharvest application of BABA at 125 mmol/L significantly suppressed green mold incidence in oranges. In this study, under room-temperature (25 ± 0.5 °C, relative humidity 80–90%) conditions, 50 mM BABA treatment effectively suppressed the incidence of postharvest anthracnose disease and the diameter expansion of lesions in mango fruits. The experimental results showed that directly inhibiting the infection of pathogenic fungus is one of the important reasons why BABA can prevent the occurrence of postharvest anthracnose disease in mango.

ROS play a dual role in the defense systems of fruits and vegetables, serving as both essential secondary messengers for physiological processes and key factors triggering oxidative damage [27,28], usually existing in the forms of O_2_^−^, H_2_O_2_ and ∙OH (O_2_^−^). When plants are infected by pathogens, ROS rapidly accumulate, leading to oxidative stress effects such as protein oxidation and denaturation, membrane lipid peroxidation, and DNA damage [29,30]. Previous studies have found that BABA is a novel inducer that can maintain the metabolic balance of ROS by regulating the antioxidant defense system in fruits [31,32,33]. The results of the present study showed that BABA treatment significantly increases the total phenolic and flavonoid content in mango (*p* < 0.05), while inducing SOD and CAT activities by 1.06-fold and 1.36-fold, respectively. At the end of storage, the O_2_^−^ production rate and H_2_O_2_ contents in the treated groups were 17.63% and 9.38% lower than those in the control group, respectively, and the DPPH radical scavenging rate increased by 13.38%. These findings align with the previous results in apples [34] and sweet berries [35], further confirming how BABA alleviates postharvest oxidative damage in fruits through the universal mechanism of activating a multi-level antioxidant defense network.

The mechanism through which BABA inhibits diseases in postharvest fruits is related to its induction of fruit defense responses. Previous studies have found that BABA can promote the synthesis of metabolites by regulating sorbitol metabolism, reactive oxygen metabolism, and phenylpropanoid metabolism [14] while inducing the production of disease-related proteins, increasing the activity of defense-related enzymes and the accumulation of reactive oxygen species, and exerting direct or indirect inhibitory effects on pathogen invasion. PAL, a pivotal enzyme in the phenylpropanoid pathway, plays an essential role in the biosynthesis of lignin, phenolic compounds, flavonoids, and salicylic acid (SA). POD contributes to cell wall formation, lignification, and mechanical strengthening against degradation, thereby safeguarding plant tissues from injury. Additionally, it catalyzes the polymerization of phenolic precursors to enhance resistance against fungal infections. Chitinase (CHI) and β-1,3-glucanase (GLU) serve as critical pathogenesis-related proteins in plant antifungal defense, countering fungal infection through the hydrolysis of chitin and β-1,3-glucans—key structural components of fungal cell walls. This study found that BABA treatment significantly increased SOD, CAT, and POD in mango, promoting the accumulation of total phenols and total flavonoids.

Correlation analysis revealed a significant negative relationship between the incidence rate, lesion diameter and the activities of SOD and CAT, and the contents of total phenols and flavonoids, indicating that BABA treatment enhances the resistance of mango fruits to anthracnose disease and increases antioxidant enzyme activity and the contents of total phenols and total flavonoids. This is consistent with the research results on BABA treatment in previous studies [36]. In addition, the incidence rate and lesion diameter were significantly positively correlated with the O_2_^−^ content, indicating that the accumulation of ROS reduces disease resistance in fruit. Thus, it is speculated that BABA treatment promotes the accumulation of phenolic and flavonoid secondary metabolites, increases the activity of antioxidant enzyme systems and the DPPH radical scavenging rate, inhibits the rate of O_2_^−^ production, reduces the active oxygen level of mango, reduces oxidative damage to the fruit, and thereby enhances the resistance of mango fruits to anthracnose disease.

The initiation of defense in fruit and vegetables is often accompanied by the expression of defense-related proteins such as PR proteins, PAL, GLU, and CHI [37,38]. Among them, the accumulation of PR proteins is correlated with pathogen-induced systemic resistance (SAR), especially PR1, which can be expressed in the SA transduction pathway. By contrast, CHI and GLU can degrade pathogen cell wall chitin and *β*-1,3-glucan [39]. The results of this study show that BABA enhances the activities of defense-response-related enzymes PAL, CHI, and GLU, as well as the relative gene expressions of disease-process-related proteins (PAL gene, GLU gene, CHI gene, and PR1 gene), promoting the production and accumulation of antimicrobial substances and effectively suppressing disease.

## 5. Conclusions

**Figure 8:**
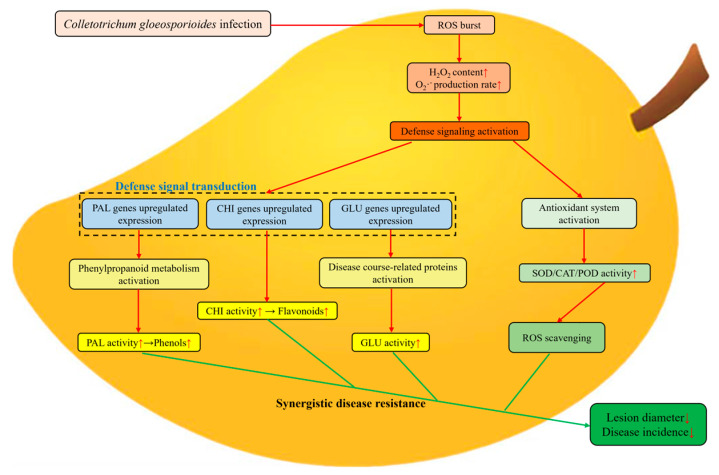
A schematic diagram of BABA-induced resistance to anthracnose in postharvest mango fruits. The red arrows inside the box indicate an increase in membrane lipid peroxidation, antioxidant enzyme activity, and secondary metabolite content; the red arrows outside the box represent positive regulation; the green arrows indicate negative regulation.

This study presents a schematic diagram of BABA-induced resistance to anthracnose in postharvest mango fruits (Figure 8). It can be deduced that BABA increases the activities of antioxidant enzymes such as SOD, CAT, and POD, as well as the contents of secondary metabolites, including total phenols and total flavonoids, thereby enhancing the DPPH radical scavenging rate. At the same time, it inhibits the production rate of O_2_^−^ and the accumulation of H_2_O_2_, improving the antioxidant capacity of mango fruits. Moreover, by inducing the activity of PAL in the phenylpropanoid metabolic pathway and the activities of disease-related proteins such as CHI and GLU, it increases the relative expression levels of PAL, GLU, CHI, and PR1 genes, enhancing the disease resistance of mango fruits. Thus, the occurrence anthracnose disease is inhibited. These findings demonstrate the strong potential of BABA as a natural and sustainable strategy for reducing postharvest losses in commercial mango storage. Its application could be integrated into existing postharvest handling systems to maintain mango quality and extend shelf life, providing an eco-friendly alternative to conventional treatments.

## Figures and Tables

**Table 1:** Sequence of primers for the target gene of mango.

Gene	Primer (Forward)	Primer (Reverse)
Actin	GTTCTACTCACTGAAGCA	CCTGGATAGCAACATACA
PAL	CTGCCAATGAGTCTGCTGTC	GAAGTTGCACCGAAACCAGT
GLU	GGACAACAACATTGGGCAGA	TTTTGGGACGTCGAGGATGA
CHI	CAGTTGCCAGTATTGTTA	TCCATCTCTTGTGTAGAA
PR1	TGCCAATCAACGTATCGGAG	CCACATCTTCACTGCATCCG

Note: Actin genes are used as internal reference genes.

**Table 2:** Correlation analysis of the incidence rate and lesion diameter of mango fruits with membrane lipid peroxidation, antioxidant enzyme, and disease-related protein.

	DIR	LD	TPC	TFC	H_2_O_2_ C	O_2_^−^ PR	DPPH RSC	SOD Activity	CAT Activity	POD Activity	PAL Activity	GLU Activity	CHI Activity
DIR	1.00												
LD	0.99 **	1.00											
TPC	−0.83 **	−0.84 **	1.00										
TFC	−0.85 **	−0.81 **	0.93 **	1.00									
H_2_O_2_ C	0.21	0.26	0.29	0.22	1.00								
O_2_^−^ PR	0.84 **	0.89 **	−0.94 **	−0.81 **	−0.04	1.00							
DPPH RSC	−0.23	−0.28	0.72 **	0.54 *	0.66 **	−0.67 **	1.00						
SOD activity	−0.79 **	−0.73 **	0.91 **	0.90 **	0.42	−0.74 **	0.55 *	1.00					
CAT activity	−0.68 **	−0.71 **	0.97 **	0.85 **	0.39	−0.93 **	0.87 **	0.81 **	1.00				
POD activity	−0.48	−0.50	0.88 **	0.78 **	0.60 *	−0.79 **	0.95 **	0.76 **	0.96 **	1.00			
PAL activity	−0.34	−0.46	0.69 **	0.46	0.27	−0.80 **	0.89 **	0.39	0.84 **	0.82 **	1.00		
GLU activity	−0.53 *	−0.59 *	0.87 **	0.66 **	0.47	−0.87 **	0.91 **	0.72 **	0.94 **	0.92 **	0.90 **	1.00	
CHI activity	−0.67 **	−0.78 **	0.52 *	0.33	−0.49	−0.77 **	0.22	0.26	0.49	0.27	0.60 *	0.53 *	1.00

Note: DIR means disease incidence rate, LD means lesion diameter, TPC means total phenol content, TFC means total flavonoid content, H_2_O_2_ C means H_2_O_2_ content, O_2_^−^ PR means O_2_^−^ production rate, and DPPH RSC means DPPH radical scavenging capacity. * and ** indicate a significant correlation at *p* < 0.05 and extremely significant correlation at *p* < 0.0l, respectively.

## Data Availability

The data are contained within the article.
